# Advances in biosensors for diagnosis and monitoring of inflammatory bowel disease: A review

**DOI:** 10.1002/btm2.70064

**Published:** 2025-08-19

**Authors:** Wenyu Fu, Ruier Xue, Mohit N. Shivdasani, Yanfang Wu, Dongfei Chen, Tianruo Guo, Nigel H. Lovell, Ewa M. Goldys, Tingxiu Xiang, Yanan Huang, Fei Deng

**Affiliations:** ^1^ Graduate School of Biomedical Engineering UNSW Sydney Sydney New South Wales Australia; ^2^ Tyree Foundation Institute of Health Engineering (iHealthE) UNSW Sydney Sydney New South Wales Australia; ^3^ Department of Chemistry University of Otago Dunedin New Zealand; ^4^ Chongqing Key Laboratory for the Mechanism and Intervention of Cancer Metastasis Chongqing University Cancer Hospital Chongqing China

**Keywords:** biomarker, biosensors, diagnosis, inflammatory bowel disease

## Abstract

Inflammatory bowel disease (IBD) encompasses a group of intestinal disorders, primarily Crohn's disease (CD) and ulcerative colitis (UC), characterized by chronic inflammation of the digestive tract. Despite extensive research, the etiology of IBD remains largely unknown, and its progression and prognosis are unpredictable, often involving uncontrolled disease behavior. Current diagnostic and monitoring techniques, such as endoscopy, scoring systems, computed tomography, and ultrasound, provide valuable tools for assessing and monitoring disease progression; but are often used in conjunction with biomarker testing to achieve rapid and accurate results. Recent advances in biosensors, which integrate biorecognition elements with signal transduction platforms, offer immense potential to improve IBD diagnostics by enabling real‐time, precise, and non‐invasive detection of biomarkers such as C‐reactive protein, calprotectin, and cytokines. This review examines existing IBD diagnostic techniques, their limitations, and the emerging role of biosensors in addressing these challenges. It explores the development of electrochemical and optical biosensors, highlights the key biomarkers utilized in these technologies, and identifies challenges and future opportunities for advancing next‐generation biosensors for IBD diagnostics and monitoring. These innovations hold promise for enhancing IBD diagnosis, monitoring, and personalized disease management.

**Translational Impact Statement**

This work highlights the emerging role of biosensors as rapid, non‐invasive tools for diagnosing and monitoring inflammatory bowel disease (IBD), addressing limitations of current clinical methods. By detecting key biomarkers such as C‐reactive protein and calprotectin with high precision, biosensors have the potential to transform IBD care through real‐time disease tracking and personalized treatment strategies.

## 1 INTRODUCTION

Inflammatory bowel disease (IBD) is a chronic recurring intestinal disorder characterized by long‐term immune‐related inflammation of the gastrointestinal (GI) tract, significantly affecting the digestive system.^1^ As a global disease, IBD has a detrimental impact on individuals' quality of life and has increasingly contributed to the global health burden in recent years.^2^ As of 2019, over 6.8 million people worldwide were diagnosed with IBD.^3^ This number continues to rise due to factors such as increasing life expectancy, urbanization, and dietary changes. The COVID‐19 pandemic has further worsened challenges for IBD patients, leading to higher morbidity and complications.^4^

IBD presents a complex clinical picture with overlapping symptoms, including abdominal pain, diarrhea, weight loss, and rectal bleeding.^5^ These symptoms vary in timing and severity between CD and UC.^1^, ^6^ CD affects the entire GI tract and involves all layers of the intestinal wall, while UC is confined to the colonic mucosa.^7^ Over 30% of IBD patients also experience extraintestinal complications, such as arthritis, uveitis, and skin disorders, which complicate diagnosis and management further.^8^ Differentiating IBD from conditions like intestinal tuberculosis (ITB) and chronic infectious colitis (IC) is particularly challenging due to similar clinical features and shifting epidemiological patterns.^9^

Traditional diagnostic approaches, including endoscopy,^10^ imaging,^11^ and other techniques,^12^, ^13^, ^14^ provide accurate insights into disease location, condition, and severity.^11^ More recently, biomarker testing has been increasingly used in conjunction with other techniques for more accurate results.^12^ However, limitations of current biomarker testing include relying on multiple tissue biopsies, patient compliance for at‐home tests, and specialized pathology labs. This limits their accessibility and practicality and limits real‐time testing as well as at point‐of‐care. To address these limitations, biosensors have emerged as a promising approach that may improve biomarker testing. These biosensing devices combine biorecognition elements with advanced signal transduction technologies to detect biomarkers like C‐reactive protein (CRP), calprotectin, and cytokines.^13^ They can analyze biological samples such as blood, stool, saliva, and urine.^14^ By enabling rapid, real‐time, and non‐invasive diagnostics, biosensors address critical gaps in current IBD management.

Most previous reviews have focused on specific aspects of IBD diagnostics and monitoring tools, such as endoscopic and imaging techniques. There is a lack of a comprehensive overview that integrates these elements and explores new emerging technologies. This review aims to fill that gap. It examines existing diagnostic approaches, discusses the limitations of current methods, and highlights the potential of biosensors to improve IBD diagnostics. By addressing these key areas, the review provides a foundation for future research and innovation in this critical field.

## 2 CURRENT DIAGNOSTIC APPROACHES AND CHALLENGES FOR INFLAMMATORY BOWEL DISEASE

**FIGURE 1:**
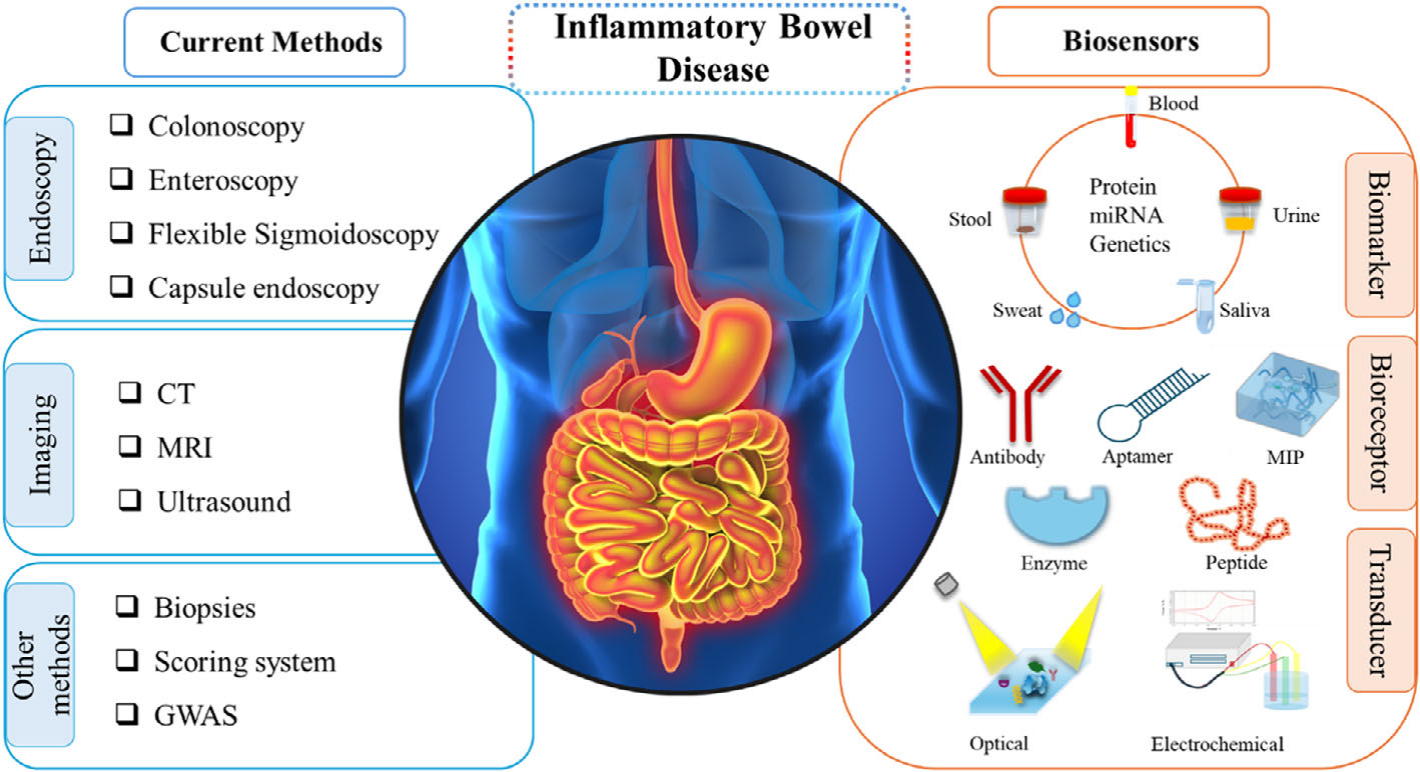
Schematic diagrams of current methods and novel biosensors that hold high promise for IBD diagnosis and monitoring. Abbreviations: CT (computed tomography), MRI (magnetic resonance imaging), GWAS (genome‐wide association studies), and CRP, MIP (molecularly imprinted polymer).

**FIGURE 2:**
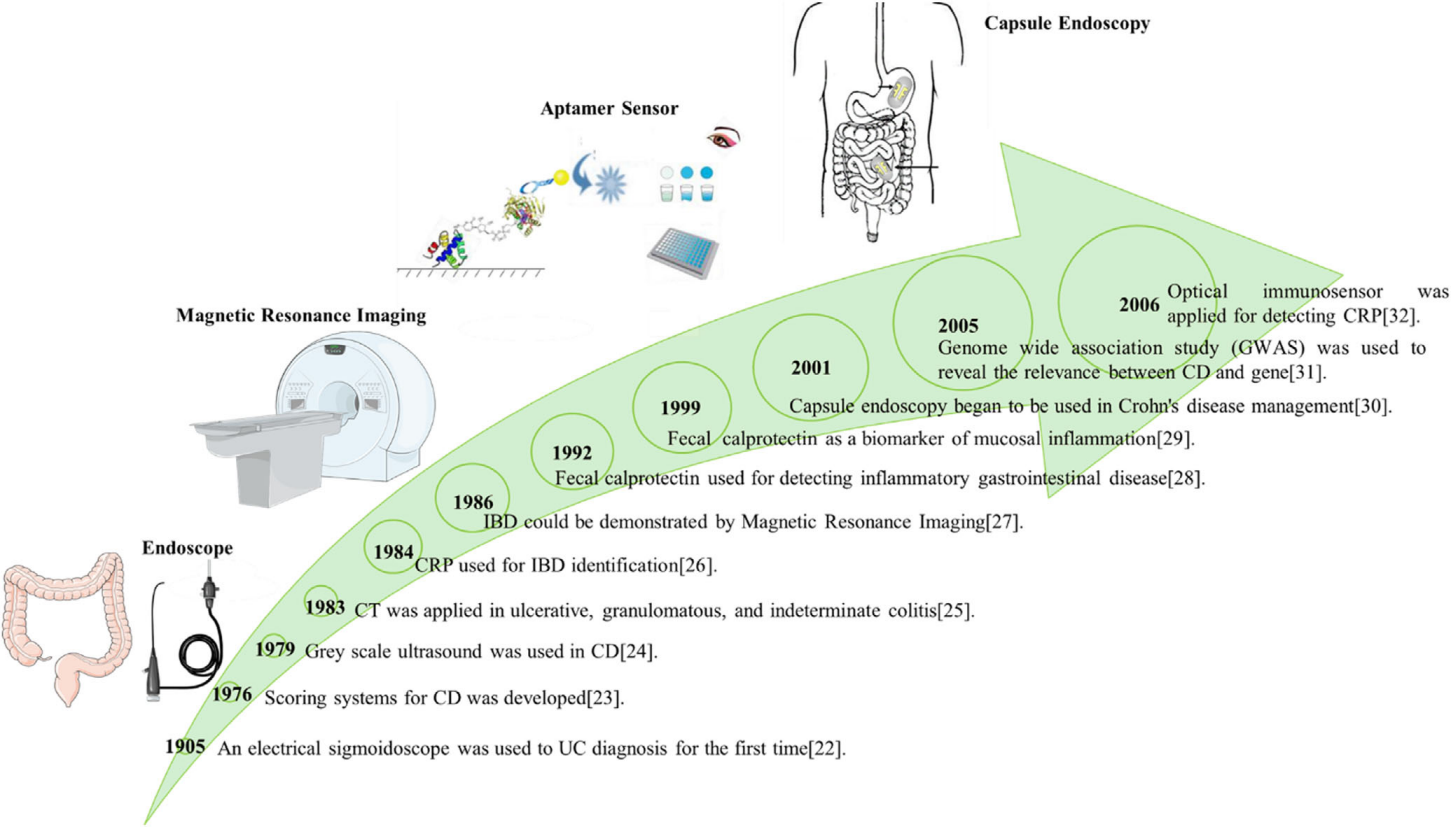
Timeline of the development of IBD diagnosis and monitoring approaches.^22^, ^23^, ^24^, ^25^, ^26^, ^27^, ^28^, ^29^, ^30^, ^31^, ^32^ The diagnostic methods initiated from endoscopy, followed by imaging, then leading to biosensors.

Current methods for identifying, diagnosing, and monitoring IBD include different endoscopy techniques,^10^, ^15^, ^16^, ^17^ imaging techniques^18^, ^19^, ^20^ and other approaches^21^ (Figure 1). The development of IBD diagnosing approaches is summarized based on the initial use of each diagnostic technique and biomarker identified as important for IBD (Figure 2). The diagnostic methods initiated from endoscopy, followed by imaging, then leading to the newest biosensors. This section provides a concise overview of these medical approaches in the early diagnosis of IBD, along with their associated limitations.

### 2.1 Endoscopy

Endoscopy plays a crucial role in diagnosing IBD, distinguishing between UC and CD, and monitoring disease activity in clinical practice.^10^, ^15^, ^16^, ^17^ The main endoscopic approaches used for IBD diagnosis include colonoscopy, flexible sigmoidoscopy, esophagogastroduodenoscopy (EGD), and wireless capsule endoscopy.^10^, ^15^, ^16^, ^17^

The first documented use of an endoscope for identifying IBD dates back to 1905 (Figure 2); when Lockhart Mummery used an electric sigmoidoscope to identify colitis with ulceration symptoms, marking a significant milestone in IBD diagnosis.^22^ Following this, with advancements in technology and the growing need for intestinal disease diagnosis, the period between 1968 and 1990 became the “golden era” of endoscopy, leading to innovations such as fiberscopes and dye‐based endoscopy in the 1970s.^33^ In the 2000s, capsule endoscopy was applied for CD diagnoses for the first time, further expanding diagnostic capabilities.^34^ The advances of endoscopic technology have since improved image clarity and diagnostic accuracy in IBD.

Colonoscopy, often combined with ileoscopy, can be used to directly visualize the colon and terminal ileum while also enabling biopsies. It is currently recommended for the evaluation of patients exhibiting indicative IBD symptoms.^10^, ^16^ Combining the results of colonoscopy and biopsy is not only useful in differentiating UC and CD, but is also helpful to exclude other bowel infections, including infection‐induced colitis, drug‐induced colitis, and ischemic colitis.^16^, ^35^, ^36^ During the procedure, at least two biopsy specimens should be collected from five different sites across the ileum and rectum for further diagnosis.^37^ The key endoscopic features of UC include mucosal granularity, erosions, and ulcers, while CD patients show “cobblestone” like mucosa, involvement discontinuously, and anal lesions.^38^ Colonoscopy with ileoscopy is generally safe for individuals, with a low incidence of adverse effects (0.7% major complications and 3.9% minor complication).^39^ However, its accuracy decreases in severe cases, with an error rate increased to 9% in severe bowel inflammations.^38^ In addition, colonoscopy is not recommended for patients with severe colitis or toxic megacolon, where an alternative method, flexible sigmoidoscopy, should be considered.^10^, ^16^

Flexible sigmoidoscopy is a cost‐effective, time‐efficient, and safe medical procedure, and it does not require sedation during the process.^40^ It is useful in assessing the disease severity and excluding ischemic colitis or infection‐induced colitis.^17^ However, it is insufficient for the assessment of isolated proximal colitis, and flexible sigmoidoscopy alone is not adequate for UC identification.^10^, ^17^

Esophagogastroduodenoscopy (EGD) is a type of upper endoscopy commonly used for suspected pediatric IBD patients, as upper gastrointestinal CD is more common in children than adults.^41^ If IBD‐suspected patients have symptoms including abdominal pain, vomiting, nausea, and weight loss, EGD should be performed.^16^ Like colonoscopy, at least two biopsy specimens should be taken from multiple sites within the esophagus, stomach, and duodenum of suspected IBD patients during EGD for better accuracy.^42^, ^43^ According to the results of EGD, ulcerations, erythema, lesions, strictures, and fistula openings are the main endoscopic features of upper gastrointestinal CD.^41^

Capsule endoscopy is a method that can directly visualize the small bowel mucosa in a minimally invasive manner.^44^, ^45^, ^46^ It has a high diagnostic yield for CD, particularly in detecting superficial lesions that traditional endoscopy might miss.^10^ However, its application is limited due to several challenges, including high false‐positive rates, which may cause overdiagnosis^47^ and low specificity (53%) in detecting small‐bowel CD,^48^ and capsule retention risk, up to 13% in suspected CD cases, because of the small bowel strictures.^49^ Therefore, the use of capsule endoscopy in CD diagnosis might need prior small bowel imaging.^49^

Overall, endoscopy remains essential for IBD diagnosis and monitoring, with colonoscopy and ileoscopy as the gold standards. Other endoscopic techniques include flexible sigmoidoscopy, EGD, and capsule endoscopy; they also offer high diagnostic value for IBD diagnosis. However, their effectiveness may be limited in severe conditions or when influenced by other bowel conditions.

### 2.2 Imaging

Imaging techniques play a crucial role in IBD diagnosis, offering non‐invasive tools to visualize and evaluate intestinal inflammation. The main techniques include computed tomography (CT), ultrasound, and magnetic resonance imaging (MRI),^18^, ^19^, ^20^ each with distinct advantages and limitations for clinical use.

CT uses X‐rays and contrast agents to enhance visualization of different body regions, aiding in disease differentiation. In 1983, CT was first applied to identify UC and CD, with researchers comparing key aspects of UC and CD, including colon wall thickness (7.8 mm in UC, 13 mm in CD), attenuation (uneven in UC, uniform in CD), and fat proliferation around the rectum in UC patients.^50^ These findings highlighted the diagnostic potential of CT for IBD. CT is particularly valued for its ability to pinpoint inflammation areas, accurately reflect changes in inflammation severity, and assess the extent of IBD.^51^ CT enterography, which uses neutral enteric contrast and a thin‐slice technique, has become the preferred approach for IBD evaluation, providing clear visualization of bowel wall enhancement, thickening, and stratification in CD cases.^52^ However, CT exposes patients to ionizing radiation, leading to health risks for patients, requiring cautious use during repeated applications.

Ultrasound was first applied to diagnose CD in 1979, providing a safer alternative to CT by avoiding ionizing radiation exposure.^24^ It is a simple, safe, inexpensive, non‐invasive, and applicable method for detecting IBD.^19^, ^53^ Different frequency probes (3–5 and 5–15 MHz) enable visualization of the five‐layer bowel wall and key intestinal regions in suspected IBD patients.^19^ Ultrasound has shown high sensitivity and specificity in diagnosing CD and detecting relevant complications, with increased bowel wall thickening and the proliferation of fibrofatty being the main features.^54^, ^55^ However, ultrasound has limited utility in initial UC diagnosis, as bowel wall stratification remains largely intact in UC cases, and mucosal changes cannot be specifically detected due to its low spatial resolution.^53^ Additionally, ultrasound results are influenced by operator expertise, device variations, and frequency settings.^53^ Moreover, it is also less effective in assessing structures situated deeply and the upper gastrointestinal tract in pediatric patients.^56^

Compared to CT, MRI has the advantage of avoiding radiation risk, which is beneficial for IBD patients requiring frequent imaging over time.^57^ MRI provides real‐time imaging with superior soft tissue contrast without the need for intravenous contrast, making it useful for detecting inflammation‐related changes, luminal and mural abnormalities, and bowel wall oedema.^57^ The most commonly used MRI technique in clinical practice is MR enterography, which provides more detailed visualization of bowel inflammation compared to CT enterography, especially in suspected CD cases.^58^ However, MRI is expensive, requires longer examination times, and has lower spatial resolution with variable image quality.^57^, ^59^

In summary, CT, MRI, and ultrasound each provide valuable, non‐invasive diagnostic tools for identifying and assessing IBD. While they offer unique benefits, their clinical utility is balanced by specific limitations, such as radiation risks associated with CT, ultrasound's operator dependency, and limitations in deeper or pediatric assessments, and MRI's high costs and variable image quality. Therefore, selecting the most appropriate imaging technique should be guided by clinical context, patient characteristics, and diagnostic requirements to optimize accuracy and patient safety in IBD management.

### 2.3 Other methods

Other options, such as scoring systems, GWAS, and biopsies, are also essential in IBD management. These methods help provide a more comprehensive understanding of IBD as a complement to clinical trials.

Scoring systems for inflammatory bowel disease (IBD) are broadly categorized into symptom‐based indices and endoscopy‐based assessments, both of which play critical roles in disease classification and monitoring therapeutic response. Symptom‐based scoring systems, such as the Crohn's Disease Activity Index (CDAI) and the Harvey‐Bradshaw Index, quantify disease activity using clinical symptoms including abdominal pain, stool frequency, and general well‐being. CDAI, introduced in 1976, requires a 7‐day record of symptoms and is influenced by variables such as patient health status, medication use, and surgical history.^23^, ^60^ The Harvey‐Bradshaw Index offers a simplified alternative, providing comparable outcomes with fewer input variables and requiring <1 day to complete.^61^ In contrast, endoscopy‐based scoring systems evaluate mucosal inflammation directly via imaging. For UC, the Mayo Endoscopic Subscore and the Ulcerative Colitis Endoscopic Index of Severity (UCEIS) are commonly used to grade disease severity based on endoscopic findings. These objective assessments are essential for evaluating mucosal healing, a key target in modern IBD management. While both types of scoring systems enhance the systematic assessment of IBD, each has limitations related to subjectivity, invasiveness, and dependence on patient or procedural factors.

GWAS have revealed the relationships between specific diseases and relevant genetic variants through statistical analysis, and they have been used for disease diagnosis and therapy.^62^ In 2001, the connection between CD and NOD2 variants was discovered; following in 2005, GWAS was first employed in an IBD study, revealing that TNFSF15 is linked to CD.^31^, ^63^ With the advancement of this technique, more loci in connection with IBD have been found and recognized by researchers.^64^ However, GWAS cannot thoroughly explain the genetic variations occurring in CD, and the precise functions of the associated variants remain unclear.^64^ Consequently, the relationship between IBD and the associated genetic variants needs to be further studied.

Biopsies, which involve the collection and analysis of patients' cells, tissues, and biofluids, are particularly effective in distinguishing IBD from non‐IBD colitis and in differentiating between UC and CD.^65^ When combined with endoscopic evaluation, biopsies remain the gold standard for accurate diagnosis and treatment planning in IBD.^66^ However, their diagnostic accuracy depends heavily on sample quality and the technical expertise of the personnel involved.^66^ Moreover, obtaining multiple biopsy samples, which is often necessary to ensure diagnostic accuracy, can be practically challenging—particularly in pediatric patients due to procedural discomfort and safety concerns. In summary, while biopsies offer high diagnostic value, proper sample handling and procedural precision are essential, and practical limitations should be considered in specific patient populations.

These supplementary diagnostic techniques, scoring system, GWAS, and biopsies offer more details for recognizing and managing IBD. The scoring systems provide standardized and continuous illness tracking; GWAS can expose the relevance of IBD and genes, and biopsies can provide cellular level evaluation for IBD management. While these methods have drawbacks, applying them in combination could promote the accuracy of diagnosing, tracking, and controlling IBD.

## 3 BIOMARKER TESTING FOR IBD

Biomarkers are quantifiable elements from biofluids, tissues, or cells that play a vital role in detection, diagnosis, and management of inflammatory disorders.^14^ Their detection and management is essential in IBD diagnosis and monitoring for many years due to their safety, minimal invasiveness, simplicity, and cost‐saving nature.^67^ These biomarkers aid clinicians in evaluating disease presence, conditions, and variance.

IBD‐related biomarkers are generally classified into three main types: protein, miRNA, and genetics. Protein biomarkers, such as CRP, calprotectin, and numerous cytokines, are widely studied, with blood and feces as the main sources. Among protein biomarkers, calprotectin from feces and CRP from blood samples are the only two clinical biomarkers that show concordance with endoscopy results and have been verified in many studies.^68^ CRP has proved to be a clinically valuable biomarker for CD cases, and calprotectin concentration change is efficient in distinguishing IBD and non‐IBD cases and is also advantageous for further treatment.^14^ Exploring other emerging biomarkers (miRNA and genetic biomarkers) is in development, some of which have been emphasized to be efficient in the IBD evaluation field at the preclinical stage.^14^ However, the data related to these emerging biomarkers still need to be clinically verified.^68^ In addition, some studies have pointed out that biomarkers cannot be applied individually for the precise diagnosis and monitoring of IBD or the differentiation of UC, CD, and other colitis, because the concentration change of a single biomarker may be caused by different impact factors.^14^ Thus, the multiplexed detection of biomarkers is an important need in IBD diagnostics and monitoring.

### 3.1 Protein biomarkers

Protein biomarkers could be used for the identification of biological changes, potentially allowing immunity, inflammation, and relevant diseases and syndromes to be detected. Table 1 provides information on clinically verified biomarkers (CRP and calprotectin) and emerging protein biomarkers (cytokines and others) for IBD.

**TABLE 1:** Clinically verified and emerging protein biomarkers for IBD.

Sample	Biomarker	Concentration	Median	Condition	Measure method	Reference
Blood	CRP	1–3 ng/mL	0.8 ng/mL	Healthy	Solid phase radioimmunoassay	^69^, ^70^, ^71^, ^72^
		0–65 ng/mL	4 ng/mL	CD (mild)	Electro‐immunoassay	
		0–15 ng/mL	0 ng/mL	UC (mild)		
		1–100 ng/mL	25 ng/mL	CD (moderate)		
		0–29 ng/mL	3 ng/mL	UC (moderate)		
		15–183 ng/mL	85 ng/mL	CD (severe)		
		2–33 ng/mL	12 ng/mL	UC (severe)		
		~200 ng/mL	–	Serum	hsCRP‐ELISA	
		25–2500 ng/mL	–	PH 7.3 PBS buffer	Magnetic immunosensor	
	Calprotectin	215.8–3770 ng/mL	1318 ng/mL	Healthy	ELISA	^73^
		410–125,000 ng/mL	8892 ng/mL	CD		
			8353 ng/mL	CD (inactive)		
			19,584 ng/mL	CD (active)		
	OSM	23.2–56.4 pg/mL	35.8 pg/mL	Healthy	CLIA	^74^
		43.3–294.4 pg/mL	121.1 pg/mL	IBD		
	TNF‐α	–	10 pg/mL	Healthy	ELISA	^75^
		–	16 pg/mL	CD		
		–	27 pg/mL	UC		
	S100A12	–	75 ng/mL	Healthy	Sandwich ELISA	^76^
		–	215 ng/mL	Inactive CD		
		–	470 ng/mL	Active CD		
		–	115 ng/mL	Inactive UC		
		–	400 ng/mL	Active UC		
	sST2	20.2–54.3 pg/mL	30.7 pg/mL	Healthy	Quantitative sandwich enzyme immunoassay	^77^
		42.7–88.4 pg/mL	63.8 pg/mL	CD		
		41.3–93.0 pg/mL	54.2 pg/mL	UC		
		25.9–68.7 pg/mL	39.9 pg/mL	IBS		
	BAFF	482–1345 pg/mL	977 pg/mL	Healthy	ELISA	^78^
		1105–1624 pg/mL	1414 pg/mL	CD		
		1018–1772 pg/mL	1417 pg/mL	UC		
	IL‐6	0.3–4.7 pg/mL	0.8 pg/mL	Healthy	ELISA	^79^
		3.7–57.3 pg/mL	10.3 pg/mL	CD		
		2.4–29.6 pg/mL	7.8 pg/mL	UC		
	IFN‐γ	0–1.67 pg/mL	0 pg/mL	Healthy	MIL‐LIPLEX MAP human cytokine/chemokine kit	^80^
		1.06–13.6 pg/mL	6.24 pg/mL	CD		
		0–6.87 pg/mL	0 pg/mL	UC		
	NGAL	–	60.06 ng/mL	Healthy	ELISA	^81^
		–	89.92 ng/mL	CD		
		–	86.62 ng/mL	UC		
	Lysozyme	3.22 ± 0.89 μg/mL	–	Healthy	Electrochemical aptamer‐based sensor	^82^
		11.9 ± 0.48 μg/mL	–	IBD		
Stool	Calprotectin	500–8000 ng/mL	2025 ng/mL	Healthy	Enzyme immunoassay	^28^
		–	43,000 ng/mL	CD		
		–	19,500 ng/mL	UC		
		–	4500 ng/mL	Healthy	ELISA	^83^
		–	31,200 ng/mL	CD		
		–	116,200 ng/mL	UC		
		20–36 μg/g	26 μg/g	Healthy	PhiCal ELISA with the new improved feces preparation	^84^
		~30,000 μg/g	–	Pancolitis		
	TNF‐α	40–84 pg/g	58 pg/g	Healthy	ELISA	^85^
		420–4322 pg/g	994 pg/g	Active CD		
		276–5982 pg/g	–	Active UC		
	S100A12	–	0.006 μg/g	Healthy	ELISA	^86^
		–	2.45 μg/g	IBD		
		–	0.05 μg/g	IBS		
	ChI3L1	–	2.2 ng/g	Healthy	ELISA	^87^
		–	18.4 ng/g	Inactive CD		
		–	632.7 ng/g	Active CD		
		–	15.8 ng/g	Inactive UC		
		–	366.6 ng/g	Active UC		
	BAFF	284–309 pg/mL	295 pg/mL	Healthy	ELISA	^78^
		326–493 pg/mL	369 pg/mL	CD		
		358–1758 pg/mL	524 pg/mL	UC		
	IL‐6	9.9–33 pg/mL	14.6 pg/mL	Healthy	ELISA	^88^
		1.6–29.9 pg/mL	11.3 pg/mL	Inactive UC		
		4.6–31.8 pg/mL	13 pg/mL	Active UC		
Sweat	TNF‐α	–	0.19 pg/mL	Healthy	Wearable sensor	^89^
		–	2.11 pg/mL	IBD		
	Calprotectin	<400 ng/mL	350 ng/mL	Healthy	Wearable biosensor	^90^
		500–1500 ng/mL	1300 ng/mL	IBD		
	CRP	–	9.33 pg/mL	Healthy	Wearable biosensor	^91^
Saliva	IL‐6	3.0–32.5 pg/mL	6.3 pg/mL	Healthy	ELISA	^79^
		2.6–300 pg/mL	16.9 pg/mL	CD		
		4.5–55.2 pg/mL	–	UC		

Abbreviations: BAFF, B‐cell‐activating factor; CD, Crohn's Disease; CHI3L1, Chitinase 3‐like‐1; CLIA, Chemiluminescence immunoassay; CRP, C‐Reactive Protein; ELISA, Enzyme Linked Immunosorbent Assay; hsCRP‐ELISA, high‐sensitivity CRP ELISA; IBD, Inflammatory bowel disease; IBS, Irritable bowel syndrome; IFN‐γ, Interferon‐gamma; IL‐6, Interleukin‐6; NGAL, Neutrophil gelatinase‐associated lipocalin; OSM, Oncostatin M; SAA, Serum Amyloid A; sST2, soluble Suppression of Tumorigenicity 2 protein; TNF‐α, Tumor necrosis factor alpha; UC, Ulcerative colitis.

#### 3.1.1 C‐reactive protein

C‐reactive Protein (CRP), an acute‐phase protein primarily synthesized by hepatocytes, is widely recognized as a valuable clinical biomarker for the diagnosis and monitoring of inflammatory bowel disease (IBD). Its concentration can increase more than 1,000‐fold within hours in response to acute inflammatory stimuli,^92^ and its relatively short half‐life of approximately 19 h enables real‐time assessment of inflammatory activity.^93^ In healthy individuals, baseline serum CRP levels typically range from 1 to 3 ng/mL, as first measured using solid‐phase radioimmunoassay in 1981.^71^ Subsequent studies, including electro‐immunoassays conducted in 1982, demonstrated the diagnostic potential of CRP in distinguishing IBD patients from healthy individuals and differentiating between CD and UC.^70^ CRP levels tend to rise more significantly in CD than in UC, making it a reliable marker for monitoring disease severity in CD. However, its utility in differentiating UC from other conditions remains limited.^70^ While CRP shows a strong correlation with UC clinical scores—except in cases of ulcerative proctitis—its correlation with CD severity is comparatively weaker.^94^ Pediatric studies have further supported these findings, showing elevated CRP levels at diagnosis in 63% of CD patients compared to only 22% of UC patients.^95^ Additionally, the CRP‐to‐albumin ratio has emerged as a promising metric for assessing IBD activity and improving differentiation between CD and UC.^96^ Despite its clinical significance, elevated CRP levels alone are insufficient to confirm UC or CD cases, as CRP is a general marker of inflammation rather than a disease‐specific biomarker for IBD.^97^ Therefore, integrating CRP quantification with other diagnostic approaches—such as fecal biomarkers, imaging, and serological tests provides— a more comprehensive and accurate strategy for IBD diagnosis and management.^14^

A variety of methods have been developed to measure CRP levels in biological samples, each offering distinct sensitivities and clinical applications. The most commonly employed clinical techniques are high‐sensitivity CRP (hsCRP) assays, typically based on nephelometry and turbidimetry.^98^ In recent advancements, a magnetic immunosensor has been introduced for CRP detection in human serum, saliva, and urine, demonstrating a linear detection range of 25–2500 ng/mL. This sensor has exhibited superior sensitivity compared to conventional hsCRP assays and shows promising potential for diagnostic use in both IBD and cardiovascular diseases.^72^ Another rapid method, the lateral flow immunochromatographic assay (LFIA), delivers results within 15 min and covers a detection range of 0.1–10 ng/mL.^99^ Additionally, a label‐free fiber‐optic surface plasmon resonance (SPR) biosensor has been developed for specific CRP detection, offering a linear response within the range of 0.01–20 μg/mL. The sensor achieved its highest sensitivity at 1.17 nm per log(μg/mL).^100^ Collectively, the established CRP biosensors have demonstrated excellent performance in laboratory settings using small volumes of biological samples. However, they have not yet been validated with large‐scale clinical sample testing, nor have they been successfully integrated with current gold standard imaging and endoscopic diagnostic methods.

#### 3.1.2 Calprotectin

Calprotectin is a protein derived from neutrophils that can bind to calcium and is associated with autoimmune activity.^101^ As shown in Table 1, fecal calprotectin levels are useful in diagnosing healthy subjects with IBD patients, with the concentration of fecal calprotectin of 2,025 ng/mL compared with 43,000 ng/mL for CD and 19,500 ng/mL for UC, which means it is useful in differentiating UC and CD.^28^ Thus, the usage of fecal calprotectin is a practical, non‐invasive clinical biomarker for IBD diagnosis, making it widely utilized in clinical settings, such as Quantum Blue® Calprotectin by Bühlmann Laboratories AG.^102^

Measurement of fecal calprotectin has evolved over time, but significant variability remains due to differences in assay techniques and sample processing. For instance, a study in 2000 introduced a modified sample preparation method and reported results in μg/g rather than the more commonly used ng/mL, suggesting an average fecal calprotectin level of 26 μg/g in healthy individuals.^84^ Subsequent research widely adopted a 50 μg/g cut‐off for distinguishing inflammatory conditions, with some studies recommending a higher threshold of 100 μg/g to improve diagnostic accuracy.^84^, ^103^, ^104^ In recent years, several novel methods—primarily based on ELISA—have been developed for fecal calprotectin quantification. However, considerable variability in results has been reported due to differences in ELISA kits, antibody specificity, and fecal sample extraction protocols.^105^, ^106^ To ensure consistency and comparability across studies and clinical settings, the development and adoption of standardized reference protocols for calprotectin extraction, preparation, and measurement are critically needed.

Although calprotectin is a valuable biomarker, it is not specific to inflammatory bowel disease (IBD) and can also be elevated in other gastrointestinal inflammatory conditions. Consequently, combining calprotectin with additional biomarkers, such as C‐reactive protein (CRP), can improve diagnostic accuracy.^107^ Beyond its diagnostic utility, calprotectin also serves as an important indicator of mucosal healing, making it a useful tool for monitoring treatment response in IBD patients.^102^

#### 3.1.3 Cytokines

Cytokines are low‐molecular‐weight proteins secreted by immune cells that play crucial roles in cell signaling, proliferation, and immune regulation, particularly in the context of chronic intestinal inflammation.^108^ Proinflammatory cytokines such as tumor necrosis factor‐alpha (TNF‐α) and interleukin‐6 (IL‐6) are strongly associated with the progression and exacerbation of inflammatory bowel disease (IBD)^108^, ^109^; highlighting their potential as clinical biomarkers for IBD diagnosis and disease monitoring.

Tumor necrosis factor‐alpha (TNF‐α) is a proinflammatory cytokine secreted by T cells, macrophages, and monocytes, playing a critical role in regulating inflammation and immune responses. While essential for infection control, excessive production of TNF‐α can impair immune function and contribute to the development of immune‐mediated diseases.^110^ The involvement of TNF‐α in inflammatory bowel disease (IBD) has been well established, with clinical studies reporting elevated levels in IBD patients—31% higher in UC and 80% higher in CD compared to healthy controls.^111^ Further investigations have quantified TNF‐α concentrations at 7.55 ng/mL in UC and 12.7 ng/mL in CD, markedly higher than the 0.02 ng/mL observed in healthy individuals, underscoring its potential as a disease‐specific biomarker.^112^ Notably, TNF‐α levels remain stable in patients with inactive CD, making it a valuable indicator for distinguishing between active and inactive disease states.^108^

Interleukin‐6 (IL‐6) is a key cytokine involved in immune‐mediated diseases, with normal concentrations ranging from 0.3 to 4.7 ng/mL in blood,^79^ 9.9 to 33 pg/mL in stool,^88^ and 3.0 to 32.5 ng/L in saliva.^79^ Primarily synthesized by T cells, IL‐6 plays a pivotal role in inducing the production of other cytokines and orchestrating immune responses.^109^ Compared with other biomarkers such as CRP and calprotectin, IL‐6 has been recommended as a promising non‐invasive indicator for IBD detection.^113^ Several studies have demonstrated that IL‐6 levels are effective in distinguishing between active (142.3–148.4 ng/mL) and inactive (114.3–116.2 ng/mL) IBD, although its ability to differentiate between UC and CD remains limited.^114^ Moreover, a positive correlation between IL‐6 and CRP levels has been observed, suggesting a synergistic role in IBD‐related inflammation.^114^ Additional research has also indicated that IL‐6 fluctuations may be linked to UC progression and its associated cancer risks.^115^

#### 3.1.4 Other protein biomarkers

In addition to clinically validated protein biomarkers such as CRP and calprotectin, several other protein biomarkers show significant potential for improving IBD diagnosis and monitoring, owing to their strong association with immune responses and inflammation.

S100A12 is a small, calcium‐binding proinflammatory protein involved in cytokine release and the regulation of inflammatory responses.^86^, ^116^ It can be detected in both blood and stool, with levels rising significantly in patients with inflammatory bowel disease (IBD).^68^, ^76^ For instance, one study reported average blood concentrations of S100A12 at approximately 75 ng/mL in healthy individuals, compared to 115–470 ng/mL in IBD patients (Table 2).^76^ Similarly, stool concentrations of S100A12 were found to be markedly elevated in active IBD cases (2.45 mg/kg) relative to healthy controls (0.006 mg/kg),^86^ highlighting its diagnostic value. Furthermore, stool‐based S100A12 has been proposed as a more effective non‐invasive biomarker than fecal calprotectin, underscoring its strong potential for clinical application in IBD detection and monitoring.^68^

**TABLE 2:** miRNA biomarkers applied in IBD.

IBD types	Sample sources	Expression	miRNAs	Ref
CD	Blood	Increase	miR‐16, miR‐23a, miR‐29a, miR‐106a, miR‐107, miR‐126, miR‐191, miR‐199a‐5p, miR‐200c, miR‐362‐3p, miR‐532‐3p	^133^
			miR‐101, miR‐375	^136^
			miR‐340	^132^
			miR‐223	^137^
			miR‐16, miR‐21, and miR‐223	^138^
			miR‐595 and miR‐1246	^139^
			miR‐199a‐5p, miR‐362‐3p, and miR‐532‐3p and miRplus‐E1271 (active CD)	^132^
		Decrease	miR‐21, miR‐31, miR‐146a, miR‐155	^136^
			miR‐149	^132^
			miR‐19a‐3p and miR‐19b‐3p	^140^
			miRplus‐F1065 (active CD)	^132^
	Biopsies	Increase	miR‐30c, miR‐130a	^141^
			miR‐146a, miR‐155	^142^
			miR‐31	^143^
			miR‐19a, miR‐31, miR‐375	^136^
		Decrease	miR‐141, miR‐200a, miR‐200b and miR‐200c	^134^
	Saliva	Increase	miR‐101	^136^
	Stool	Increase	miR‐16‐5p	^144^
			miR‐15a‐5p, miR‐16‐5p, miR‐128‐3p, miR‐142‐5p, miR‐24‐3p, miR‐27a‐3p, miR‐223‐3p, miR‐223‐5p, and miR‐3074‐5p	^145^
			miR‐155 and miR‐223	^138^
		Decrease	miR‐10a‐5p, miR‐10b‐5p, miR‐141‐3p, miR‐192‐5p, miR‐200a‐3p, miR‐375, miR‐378a‐3p	^145^
UC	Blood	Increase	miR‐16, miR‐21, miR‐28‐5p, miR‐151‐5p, miR‐155, miR‐199a‐5p	^133^
			miR‐16, miR‐21, and miR‐223	^138^
			miR‐19a, miR‐101, miR142‐5p, miR‐223, miR‐375, and miR‐494	^136^
			miR‐103‐2, miR‐362‐3p	^132^
			miR‐223	^137^
			hsa‐miR‐4454, hsa‐miR‐223‐3p, hsa‐miR‐23a‐3p, and hsa‐miR‐320e	^135^
			miR‐595 and miR‐1246	^139^
		Decrease	miR‐505	^132^
			miR‐21, miR‐31, and miR‐146a	^136^
	Biopsies	Increase	miR‐31‐3p	^146^
			miR‐15	^147^
			miR‐125b, miR‐223	^148^
			miRNA‐206	^149^
		Decrease	miR‐141, miR‐200a, miR‐200b and miR‐200c	^134^
			miR‐26, miR‐142‐3p, and miR‐223	^136^
			miR‐142‐5p	^136^
			miR‐214‐3p	^150^
	Saliva	Increase	miR‐21, miR‐31, and miR‐142‐3p	^136^
		Decrease	miR‐142‐5p	^136^
	Stool	Increase	miR‐16‐5p, miR‐21‐5p	^144^
			miR‐155 and miR‐223	^138^

Soluble ST2 (sST2) is an isoform of the Suppression of Tumorigenicity 2 protein, which plays a significant role in various inflammatory and chronic diseases, including inflammatory bowel disease (IBD).^117^ Elevated levels of sST2 in the mucosa and serum of UC patients have been linked to disease severity and activity. For instance, active UC cases exhibit serum sST2 concentrations of 235.8 pg/mL, which are significantly higher than the 33.19 pg/mL observed in inactive cases.^118^ However, the relationship between sST2 and CD remains to be further explored.

Orosomucoid (ORM) is a low molecular weight glycoprotein that serves as an acute‐phase marker for inflammation‐related diseases, detectable in both blood and urine.^119^, ^120^, ^121^ Blood‐based ORM exhibits excellent specificity (100%) in the diagnosis of CD, though its sensitivity is moderate.^120^ In contrast, urinary ORM shows higher sensitivity and holds potential for monitoring pediatric CD cases, particularly in distinguishing between active and inactive disease states.^121^

Serum amyloid A (SAA) is synthesized in the liver and can increase by more than 1,000‐fold during inflammation, infection, or cancer.^122^ In distinguishing between active and inactive IBD, SAA expression demonstrates significant variability, with levels of 151 μg/mL in active cases and 33 μg/mL in inactive cases.^123^

B‐cell activating factor (BAFF) is a tumor necrosis factor family protein that plays a crucial role in immune‐mediated conditions.^124^ BAFF levels rise during immune responses, and fecal BAFF exhibits high sensitivity (85%–90%) in differentiating IBD from healthy samples, surpassing serum BAFF, which has a sensitivity range of 50%–64%.^78^ Additionally, both blood‐ and stool‐derived BAFF demonstrate high specificity (over 93%), indicating that BAFF could serve as a promising biomarker for the diagnostic differentiation of IBD.^78^

Although these protein biomarkers are not commonly used in clinical trials, their application could provide valuable contributions to IBD diagnosis and the development of IBD sensors.

### 3.2 Genetic biomarkers

Genetic biomarkers, which are DNA sequences from chromosomes with recognized loci, play a crucial role in the examination and evaluation of genetic diseases. In 1996, researchers identified a potential risk locus for CD on chromosome 16.^125^ Later, in 2001, the NOD2 gene (nucleotide‐binding oligomerization domain containing 2), located on this chromosome, was confirmed as a susceptibility gene for CD.^126^ The advent of GWAS in 2005 revolutionized IBD research, enabling the identification of additional genetic variants associated with the disease.^31^ For instance, variations in the TNFSF15 gene have been linked to increased risk for both CD and UC.^31^

Further studies have demonstrated that the type and occurrence of IBD may be influenced by various genes, highlighting the potential of genetic markers in IBD diagnosis and management.^127^, ^128^ For example, recent findings suggest that certain genetic factors, such as Angiotensinogen (AGT), C‐C motif chemokine receptor 7 (CCR7), G protein subunit beta 4 (GNB4), G protein subunit gamma 11 (GNG11), and Phosphoinositide 3 kinase regulatory subunit 3 (PIK3R3), are more strongly associated with UC than CD, suggesting their utility in differentiating between these conditions.^129^ Additionally, genes SLC6A14 (upregulated) and AQP8 (downregulated) exhibit the most significant expression changes in both UC and CD.^129^

By enhancing diagnostic precision, genetic biomarkers offer promising pathways for advancing personalized treatment approaches in IBD.

### 3.3 miRNA biomarkers

miRNAs are short (22 nucleotides), non‐coded RNAs that play a significant role in controlling the immune system, and the dysfunction of which might lead to immune‐related diseases.^130^ The connection between miRNA expression and IBD activities has been clarified and verified in recent years.^131^ The sample sources of miRNAs could be from blood, biopsy, saliva, and stool; the expression of which could be enhanced or reduced during the periods of having IBD (Table 2). One comprehensive research report compares and summarizes the function of miRNAs from blood samples, finding that they can be used to identify UC and CD, and determines whether they are active types.^132^ A study pointed out that quantifying miRNAs could differentiate UC and CD, which indicates that miRNAs could be functional and potentially invasive biomarkers in monitoring IBD.^133^ This research also indicated that the expression of miR‐155 is the most significant among all the samples from UC cases. Another study found that miR‐141 is efficient in identifying UC because of its overexpression.^134^ It is worth noting that some UC‐related miRNA was found more sensitive and specific than CRP.^135^

## 4 EMERGING BIOSENSORS FOR IBD DIAGNOSIS AND MONITORING

**FIGURE 3:**
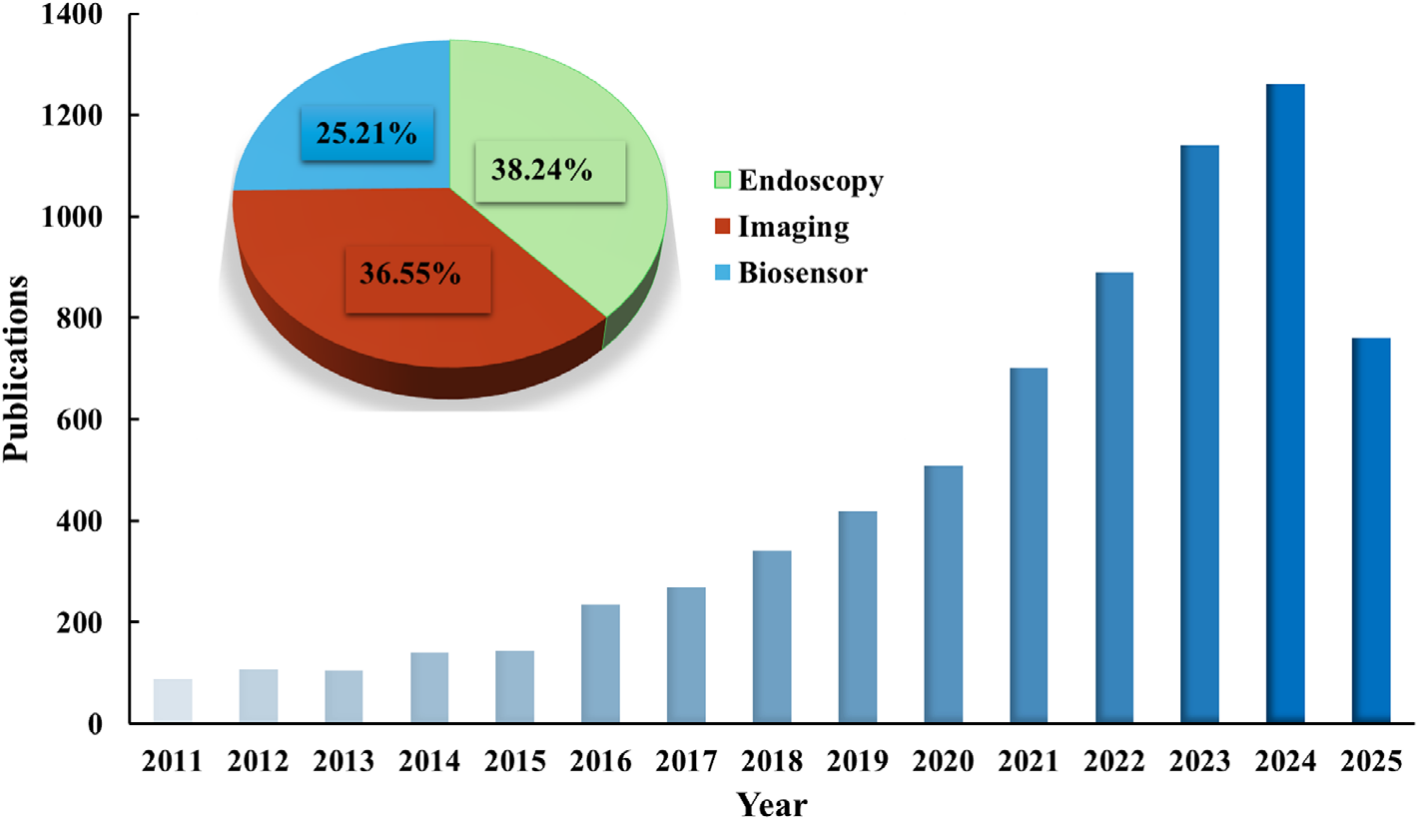
Trends in annual publications related to IBD biosensors from 2011 to 2025, based on Google Scholar search results using the terms “IBD,” “biosensor,” “biomarker,” and “diagnosis.” Publications on IBD biosensors comprised 25.21% of all IBD diagnostic research publications over this period, with a marked increase in recent years. *Data retrieved from Google Scholar (accessed June 2025)*.

Biosensors are analytical devices engineered to detect biological targets by integrating a bioreceptor with a signal transduction platform.^13^ Bioreceptors, which specifically recognize target molecules, may include aptamers, enzymes, antibodies, peptides, and molecularly imprinted polymers.^13^ Upon recognition, the resulting signal is converted into measurable outputs using transduction methods—primarily electrochemical techniques (e.g., impedimetric, voltammetric, amperometric, potentiometric) or optical techniques (e.g., fluorescence, absorbance, surface plasmon resonance, fiber optics).^151^ Recent trends show a marked increase in research focused on IBD biosensors, as illustrated in Figure 3, highlighting their growing importance in addressing key diagnostic challenges. This section examines recent advancements and diverse designs of IBD biosensors, showcasing their potential for improving disease diagnosis and monitoring.

### 4.1 Electrochemical biosensors

Electrochemical biosensors are practical and versatile tools for quantifying biological substances, offering advantages such as high efficiency, selectivity, sensitivity, and cost‐effectiveness.^152^ In constructing an electrochemical biosensor, bioreceptors are immobilized onto electrode surfaces, enabling the conversion of biological recognition events into quantifiable electrical signals, such as current.^153^, ^154^ The core mechanism of these sensors is based on redox reactions, where electron transfer occurs between the analyte, the solution, and the electrode surface under an applied external voltage.^154^ The external voltage can be applied using various electrochemical techniques for current quantification, including electrochemical impedance spectroscopy (EIS), cyclic voltammetry (CV), differential pulse voltammetry (DPV), linear sweep voltammetry (LSV), square wave voltammetry (SWV), alternating current voltammetry (ACV), and amperometry.^154^ Each technique offers specific advantages in terms of sensitivity, selectivity, and stability, and can be tailored to suit a range of applications, including the detection of IBD biomarkers. Electrochemical immunosensors, aptasensors, and peptide‐based biosensors have been developed to target different IBD biomarkers as well as biomarkers in other inflammatory conditions. Table 3 presents an overview of these three types of electrochemical biosensors.

**TABLE 3:** Emerging electrochemical biosensors for biomarker detection in IBD and other inflammatory conditions.

Electrochemical biosensor type	Bioreceptor	Biosensor structure	Sensing type	Biomarker	Sample	Detection range	Limit of detection	Disease type	Reference
Label‐free immunosensor	Antibody	Au‐SAM‐OSMab‐BSA‐OSM immunosensor	EIS CV	OSM	Blood	37–1000 pg/mL	2.86 pg/mL	IBD	^155^
		SPE‐ZnO‐anti‐calprotectin immunosensor	EIS	Calprotectin	Sweat	0.1–10 μg/mL	0.1 μg/mL	IBD	^90^
		SPE‐ZnO‐anti‐IL‐1β/anti‐CRP immunosensor	EIS	IL‐1β	Sweat	0.2–200 pg/mL	0.2 pg/mL	IBD	^91^
			EIS	CRP	Sweat	1–10,000 pg/mL	1 pg/mL	IBD	^91^
		Au‐SAM‐anti‐IL‐8 immunosensor	EIS	IL‐8	Blood	900 fg/mL−900 ng/mL	90 fg/mL	Inflammation	^156^
		Au‐SAM‐anti‐TNF immunosensor	EIS	TNF‐α	Sweat	1–200 ng/mL	1 ng/mL	Chronic diseases	^157^
		CB/PLA‐AuNPs‐Th_2_CA‐anti‐TNF‐α immunosensor	EIS CV DPV	TNF‐α	Feces	160–1820 pg/mL	44.5 pg/mL	Inflammation and bowel disorders	^158^
Label‐free aptasensor	Aptamer	GNPE‐Cys‐Avidin‐Aptamer modified biosensor	CV EIS	Calprotectin	Stool	–	5.57 μg/g	CD	^159^
		VA‐NCNTs‐Aptamer modified biosensor	CV DPV	Lysozyme	Blood	–	1.43 pg/mL	IBD	^82^
		SPE‐AuNP/GO‐COOH Aptamer modified biosensor	DPV SWV	CRP	–	0.001 ng/mL −100 ng/mL	–	Cardiovascular diseases	^160^
		Au‐aptamer/MXene modified‐dual‐target‐biosensor	EIS	TNF‐α	Blood	1 pg/mL‐10 ng/mL	0.25 pg/mL	Inflammation	^161^
		PEDOT:PSS‐AuNPs‐aptasensor	CV EIS	IL‐6	Blood	10 pM‐500 nM	576 pg/mL	Inflammation, cancer and covid‐19	^162^
		SPE‐Ti_3_C_2_T_x_/MoS_2_/Au NPs‐aptasensor	CV EIS	IL‐6	–	5 pg/mL−100 ng/mL	2.9 pg/mL	Acute inflammatory responses, chronic diseases	^163^
Competitive immunosensor	Antibody	HCPE‐ERGO‐AuPdNPs‐anti‐IL‐6 immunosensor	EIS CV LSV	IL‐6	–	0.1–100,000 pg/mL	0.006 pg/mL	Tumor diseases	^164^
Sandwich immunosensor	Antibody	SPCE‐anti‐IFN‐IFN‐biotin‐anti‐IFN immunosensor	Amperometry	IFN‐γ	Saliva	2.5–2000 pg/mL	1.6 pg/mL	Inflammatory and immune diseases	^165^
		GCE‐AuNPs‐MWCNTs‐Ab1‐BSA‐Calprotectin‐PtNi@Cu‐TCPP(Fe)‐Ab2‐BSA immunosensor	EIS CV Chronoamperometry	Calprotectin	Blood	200 fg/mL −50 ng/mL	137.7 fg/mL	Inflammatory bowel disease, hepatopathy, autoimmune disease and other inflammatory disease	^166^
Sandwich aptasensor	Aptamer	SPCEs‐magnetic particles coated with streptavidin‐aptamer‐CRP‐anti‐CRP biosensor	DPV	CRP	Blood	–	0.2 mg/L	Acute inflammatory diseases	^167^
Peptide‐based biosensor	Peptides	Au‐Ag@MoS_2_/rGO nanocomposite electrode‐peptide modified biosensor	SWV CV EIS	IL‐1β	Blood	0–250 ng/mL	42 pg/mL	Inflammatory diseases	^168^
		Au‐AuNPs@BP@PDA nanocomposite electrode‐peptide modified biosensor	SWV CV EIS	CRP	–	0–0.036 μg/mL	0.7 ng/mL	Heart and bowel inflammatory diseases	^169^
		Au‐AuNPs‐MXene‐streptavidin‐peptide modified biosensor	SWV CV EIS	Cathepsin B	Blood	–	4.5 pg/mL	Cancer and inflammatory bowel diseases	^170^

Abbreviations: ACV, alternating current voltammetry; AuNPs, gold nanoparticles; AuPdNPs, gold–palladium nanoparticles; BP, black phosphorus; BSA, bovine serum albumin; CB/PLA, carbon black/polylactic acid; CRP, C‐Reactive Protein; CV, cyclic voltammetry; Cys, cysteamine; DPV, differential pulse voltammetry; EIS, electrochemical impedance spectroscopy; ERGO, electrochemically reduced oxide; GCE, glassy carbon electrode; GNPE, gold nanoparticles electrode; GO‐COOH, carboxylated graphene oxide; HCPE, heated carbon electrode; LSV, linear sweep voltammetry; MoS_2_, molybdenum disulfide; MWCNTs, multi‐walled carbon nanotubes; OSM, Oncostatin; OSMab, Oncostatin M antibody; PDA, polydopamine; PEDOT:PSS, poly‐doped with polystyrene sulfonate; rGO, reduced graphene oxide; SAM, self‐assembled monolayer; SPCE, screen printed carbon electrode; SPE, screen printed electrode; SWV, square wave voltammetry; TCPP, Tetra (4‐carboxyphenl) porphyrin chloride; Th_2_CA, thiophene‐2‐carboxylic; VA‐NCNTs, vertically aligned nitrogen‐doped carbon nanotubes; ZnO, zinc oxide.

#### 4.1.1 Electrochemical immunosensors

Electrochemical immunosensors, which use antibodies as bioreceptors to convert antigen–antibody interactions into measurable electrical signals, have shown great potential for identifying and monitoring IBD‐related biomarkers.^155^ These sensors can be classified into label‐free,^90^, ^171^ competitive^164^ and sandwich^165^ formats.

**FIGURE 4:**
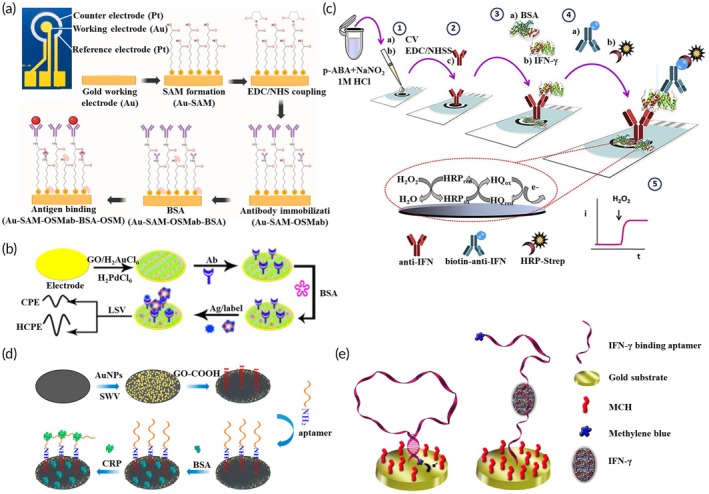
Examples of emerging electrochemical biosensors. (a) label‐free immunosensor^155^; (b) competitive immunosensor, reproduced with permission from,^164^ Copyright 2014 Elsevier; (c) sandwich immunosensor, reproduced with permission from,^165^ Copyright 2020 Elsevier; (d) label‐free aptasensor^160^; (e) methylene blue tagged aptasensor, reproduced with permission from,^174^ Copyright 2013 Elsevier.

A label‐free electrochemical immunosensor (Figure 4a) was developed for the detection of Oncostatin M in human blood, with a detection range of 37–1000 pg/mL and a limit of detection (LOD) of 2.86 pg/mL.^155^ In this study, a commercially available human serum sample was used, with the addition of interfering biomarkers such as interleukin‐1β (IL‐1β), TNF‐α, and C‐reactive protein (CRP)—all relevant to IBD—to simulate the serum composition of IBD patients.^155^ The results demonstrated that the sensor exhibited high sensitivity and selectivity for oncostatin M detection. Given these characteristics, this sensor holds promise as a potential diagnostic tool for IBD. Additionally, advances in wearable electrochemical immunosensing, when combined with a portable reader, enable continuous, non‐invasive monitoring of IBD biomarkers in sweat, as summarized in Table 3.

Advancements in fabrication techniques, such as 3D printing, have further enhanced sensor performance. A notable example is a 3D‐printed immunosensor that demonstrated strong selectivity for TNF‐α, with a limit of detection (LOD) of 44.5 pg/mL, highlighting its potential for detecting inflammatory biomarkers.^158^ Similarly, the use of novel materials—such as graphene and metal nanoparticles—has significantly improved sensor sensitivity. For instance, a competitive immunosensor for IL‐6 detection utilized electrochemically heated electrodes to enhance analytical performance.^164^ As illustrated in Figure 4b, the transducer platform was constructed from nanoscale composite materials, including graphene, gold, and palladium nanoparticles, which provided excellent biocompatibility and contributed to improved sensor functionality.^164^

Sandwich immunosensors have also benefited from the integration of advanced materials. For example, a Cu‐TCPP(Fe)‐based biosensor designed for calprotectin detection achieved an ultrasensitive limit of detection (LOD) of 137.7 fg/mL.^166^ In a similar study, a sandwich amperometric immunosensor was developed to detect IFN‐γ in saliva. In this design, the capture antibody (anti‐IFN) was immobilized on the electrode surface, while the detection antibody (biotin‐anti‐IFN) was conjugated with streptavidin–horseradish peroxidase, as illustrated in Figure 4c.^165^ Notably, this biosensor required only a 5 μL sample—substantially less than the 100 μL typically needed for ELISA kits.^165^ Additionally, the detection time was reduced by 2 h compared to ELISA, and the sensor demonstrated a broader detection range (2.5–2000 pg/mL) versus the ELISA kit (15.6–1000 pg/mL).^165^ These advantages underscore the biosensor's simplicity, speed, and enhanced sensitivity, making it a promising alternative to conventional ELISA assays.

In summary, advancements in electrochemical immunosensors—including the use of novel materials, wearable formats, and innovative fabrication techniques—highlight their strong potential as efficient tools for the diagnosis and management of IBD.

#### 4.1.2 Electrochemical aptasensors

Aptamer‐based biosensors offer a cost‐effective, rapid, and reliable method for detecting IBD‐related biomarkers.^172^ These biosensors utilize aptamers, which are short RNA or single strand DNA strands, as bioreceptors to generate stable and sensitive signals for biomarker monitoring.^173^ A notable example is a label‐free aptasensor developed for calprotectin quantification, which delivered results within 900 s and achieved a limit of detection (LOD) of 5.57 μg/g. Its performance was validated against lateral flow tests, highlighting its potential for CD diagnosis.^159^

Advances in electrode design have further improved aptasensor performance for other possible biomarkers (Table 3). For instance, a voltammetric aptasensor using vertically aligned nitrogen‐doped carbon nanotube carpet electrodes showed enhanced sensitivity and electron transfer for detecting lysozyme in diluted serum samples from both IBD patients and healthy individuals.^82^ Comparison with turbidometric assay results demonstrated a strong correlation: for healthy individuals, lysozyme concentrations were 3.36 ± 0.45 μg/mL via turbidometric assay and 3.22 ± 0.89 μg/mL via aptasensor; for IBD patients, values were 11.2 ± 0.98 and 11.9 ± 0.48 μg/mL, respectively.^82^ These findings confirm the aptasensor's reliability and its promise as an effective alternative to conventional methods for lysozyme quantification in IBD diagnostics.

The integration of nanomaterials has further enhanced the performance of aptasensors. As illustrated in Figure 4d, a sensor incorporating gold nanoparticles and carboxylated graphene oxide on screen‐printed electrodes, functionalized with a CRP‐specific aptamer, demonstrated a wide detection range, stable performance over 7 days, and high sensitivity when measured using differential pulse voltammetry (DPV).^160^ This improved detection capability highlights the advantages of employing advanced nanomaterials.^160^ Electrothermal strategies have also contributed to greater sensor efficiency. For instance, an aptasensor utilizing alternating current electrothermal flow enabled simultaneous detection of TNF‐α and IFN‐γ within 10 min, achieving detection limits of 0.25 and 0.26 pg/mL, respectively (Table 3).^161^ The use of gold microgap electrodes integrated with MXene nanosheets further amplified the electrical signals and enhanced overall conductivity.^161^

Signal amplification strategies have demonstrated significant effectiveness in enhancing aptasensor performance. For example, an IFN‐γ‐specific aptamer labeled with methylene blue facilitated improved electron transfer; thereby increasing sensitivity and overall detection efficiency. This configuration, illustrated in Figure 4e, highlights the critical role of innovative signal‐enhancing techniques in biosensor design.

In summary, aptasensors show considerable potential for the detection and monitoring of IBD biomarkers. Label‐free configurations offer simple and rapid detection, while advances in electrode architecture and incorporation of nanomaterials contribute to improved sensitivity and signal stability. Moreover, the integration of electrothermal methods and signal amplification strategies further optimizes sensor functionality, positioning aptasensors as powerful tools in the diagnostic landscape of IBD.

### 4.2 Optical biosensors

Optical biosensors are valuable tools for detecting inflammatory biomarkers, as they convert biological interactions into quantifiable light‐based signals upon the presence of target biomarkers.^175^ These sensors enable rapid biomarker identification with high selectivity and sensitivity, making them highly suitable for inflammation monitoring.^75^ However, research on the application of optical biosensors specifically for IBD diagnosis remains limited. While some optical biosensors have been developed for detecting inflammation‐related biomarkers, they are not yet tailored specifically for IBD detection.

**FIGURE 5:**
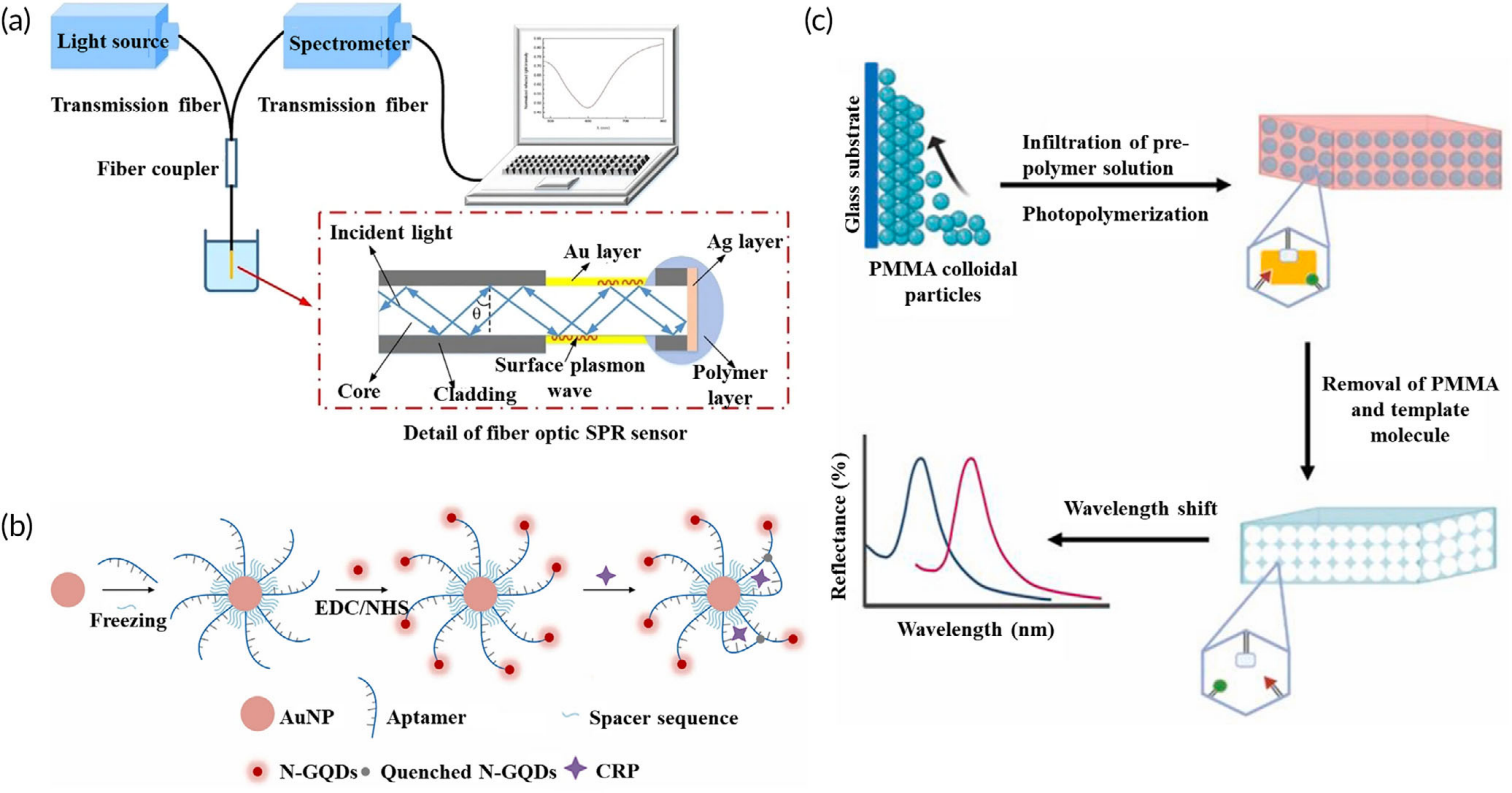
Examples of emerging optical biosensors. (a) Label‐free fiber optic SPR biosensor for CRP quantification, reproduced with permission from^100^; (b) Fluorescent aptasensor for CRP detection, reproduced with permission from,^179^ Copyright 2022 Elsevier; (c) Molecularly imprinted photonic hydrogel‐based optical sensor used for calprotectin detection, reproduced with permission from,^180^ Copyright 2023 Elsevier.

SPR‐based biosensors have emerged as valuable tools for detecting disease biomarkers due to their multifunctionality, cost‐effectiveness, high sensitivity, and stability.^176^ For instance, a label‐free SPR immunosensor designed for CRP detection (Figure 5a) demonstrated a linear detection range of 0.01–20 μg/mL, with optimal sensitivity at 1.17 μg/mL.^100^ This disposable and easy‐to‐fabricate sensor minimizes cross‐contamination risks,^100^ making it a potential tool for clinical applications and a potential candidate for IBD detection in the future. However, it is important to note that the study referenced only tested the SPR biosensor in CRP solutions of varying concentrations diluted in PBS, rather than in real human or animal samples. In addition, although the sensor showed specificity for CRP, the study did not compare CRP concentrations in disease states, including IBD. Further research is necessary to validate its performance in clinical samples and assess its diagnostic potential for IBD.

Fluorescence‐based optical biosensors are widely used due to their high accuracy and sensitivity. These sensors function by absorbing short‐wavelength light and emitting longer‐wavelength light.^177^, ^178^ Advances in fluorescent materials, such as graphene quantum dots (GQDs), have further improved sensor performance due to their excellent biocompatibility and light stability.^179^ As shown in Figure 5b, a novel fluorescence‐based aptasensor was developed for the measurement of CRP, tested in PBS solution and acute myocardial infarction patient serum.^179^ While this sensor was designed for cardiovascular disease, it provides a valuable foundation for developing similar optical biosensors for IBD, as CRP is also a key inflammatory biomarker in IBD.

Innovative designs, such as molecularly imprinted hydrogels integrated with photonic crystals, have demonstrated significant potential for IBD diagnostics.^180^ A sensor developed for calprotectin detection in blood achieved an impressive limit of detection (LOD) of 0.07 ng/mL, exhibiting excellent linearity.^180^ This sensor utilizes PMMA particles to form a photonic structure, which, after imprinting, transforms into a hydrogel network capable of converting biological information into detectable optical signals. The binding of the target protein (calprotectin) induces structural changes in the hydrogel, generating optical signals that offer high selectivity and reliability for IBD management (Figure 5c).^180^

In summary, optical biosensors—such as SPR‐based, fluorescence‐based, and hydrogel‐photonic systems—hold great promise as platforms for the detection and monitoring of IBD biomarkers, offering significant potential for enhancing the diagnosis and management of the disease in the future.

## 5 FUTURE PERSPECTIVES

Despite significant progress in developing biosensors for IBD diagnostics, several critical research challenges persist. A primary limitation remains the relatively narrow range of clinically validated biomarkers. Currently, diagnosis relies heavily on biomarkers such as calprotectin and CRP, underscoring the need for exploring and validating additional markers, such as genetic markers, cytokine profiles, microbiome‐derived metabolites, and miRNAs, which could enhance the precision and comprehensiveness of IBD diagnostics, particularly in distinguishing between closely related gastrointestinal conditions like ITB or infectious colitis. The second gap is the clinical validation of existing electrochemical and optical biosensors. While many electrochemical biosensors have been developed for IBD diagnosis and monitoring, many have not been adequately tested against gold‐standard methods, such as endoscopy, imaging, or ELISA, nor validated in relevant clinical samples from IBD patients. Future studies should emphasize direct comparisons with traditional diagnostic techniques and utilize real clinical samples to enhance biosensor reliability and translational potential. Additionally, some investigations rely on PBS or spiked biomarker solutions rather than human or animal samples, limiting their clinical relevance. Furthermore, although wearable sensors present promising capabilities for continuous and non‐invasive monitoring, the diagnostic relevance of biomarkers detected in sweat or saliva still requires thorough evaluation against established serum or fecal markers.

Looking ahead, point‐of‐care biosensors—such as lateral flow assays (LFAs)—are the most likely to achieve clinical implementation in the near term. LFAs offer rapid, low‐cost, instrument‐free detection with visual or smartphone‐assisted readouts,^181^ making them particularly suitable for decentralized IBD testing in primary care settings, remote communities, and at‐home monitoring. Several commercial LFA‐based calprotectin tests are already in clinical use, underscoring their feasibility and patient acceptability. Compared to electrochemical biosensors, LFAs offer distinct advantages in manufacturability, usability, and regulatory readiness. Future efforts should prioritize enhancing the sensitivity and multiplexing capability of LFAs, validating performance with real‐world clinical samples, and integrating these tools into patient‐centered care pathways. Such developments will be critical for enabling early diagnosis, personalized disease monitoring, and equitable access to IBD care.

## AUTHOR CONTRIBUTIONS

Wenyu Fu: investigation; writing—original draft. Ruier Xue: investigation; writing—original draft. Mohit N. Shivdasani: writing—review & editing. Yanfang Wu: writing—review & editing. Dongfei Chen: writing—review & editing. Tianruo Guo: writing—review & editing. Nigel H. Lovell: writing—review & editing. Ewa M. Goldys: writing—review & editing. Tingxiu Xiang: writing—review & editing. Yanan Huang: conceptualization; supervision; writing—review & editing. Fei Deng: conceptualization; supervision; writing—review & editing.

## CONFLICT OF INTEREST STATEMENT

The authors have no conflicts of interest to declare.

## Data Availability

The data that support the findings of this study are available from the corresponding author upon reasonable request.
